# Harnessing the Power of ChatGPT in Cardiovascular Medicine: Innovations, Challenges, and Future Directions

**DOI:** 10.3390/jcm13216543

**Published:** 2024-10-31

**Authors:** Marc Leon, Chawannuch Ruaengsri, Glenn Pelletier, Daniel Bethencourt, Masafumi Shibata, Manuel Quiroz Flores, Yasuhiro Shudo

**Affiliations:** Department of Cardiothoracic Surgery, Stanford University School of Medicine, 300 Pasteur Drive, Falk CVRB, Stanford, CA 94305, USA; noot@stanford.edu (C.R.); gjpell@stanford.edu (G.P.); dmbcourt@stanford.edu (D.B.); quiroz33@stanford.edu (M.Q.F.)

**Keywords:** cardiovascular diseases, artificial intelligence, large language models, ChatGPT

## Abstract

Cardiovascular diseases remain the leading cause of morbidity and mortality globally, posing significant challenges to public health. The rapid evolution of artificial intelligence (AI), particularly with large language models such as ChatGPT, has introduced transformative possibilities in cardiovascular medicine. This review examines ChatGPT’s broad applications in enhancing clinical decision-making—covering symptom analysis, risk assessment, and differential diagnosis; advancing medical education for both healthcare professionals and patients; and supporting research and academic communication. Key challenges associated with ChatGPT, including potential inaccuracies, ethical considerations, data privacy concerns, and inherent biases, are discussed. Future directions emphasize improving training data quality, developing specialized models, refining AI technology, and establishing regulatory frameworks to enhance ChatGPT’s clinical utility and mitigate associated risks. As cardiovascular medicine embraces AI, ChatGPT stands out as a powerful tool with substantial potential to improve therapeutic outcomes, elevate care quality, and advance research innovation. Fully understanding and harnessing this potential is essential for the future of cardiovascular health.

## 1. Introduction

**Figure 1:**
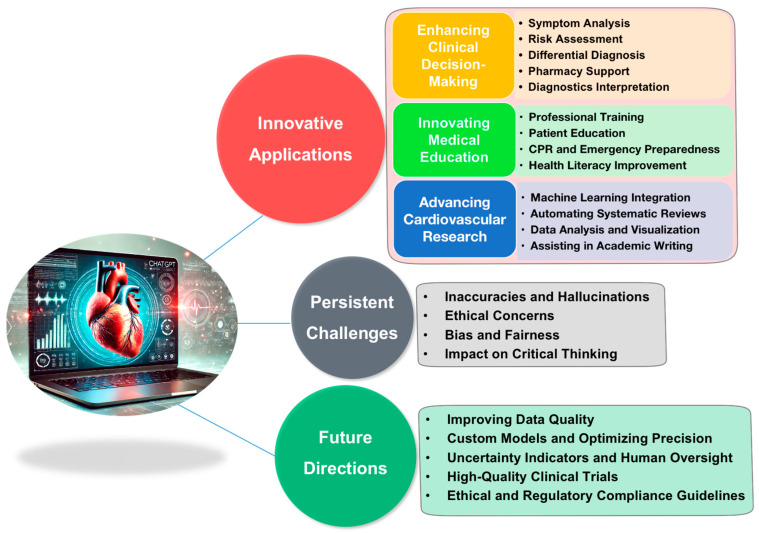
ChatGPT in cardiovascular medicine. This graphical abstract summarizes the article’s overview of ChatGPT’s innovative applications in cardiovascular medicine, its persistent challenges, and future directions for optimizing its role and effectiveness.

The rapid advancement of artificial intelligence (AI) is profoundly transforming the medical field, revolutionizing clinical practices, patient care, medical education, and research methodologies [1,2]. Among various AI technologies, large language models (LLMs) such as ChatGPT are particularly noteworthy due to their broad applicability, extensive public recognition, and widespread adoption [3,4,5]. Cardiovascular diseases (CVDs) represent the most prevalent and severe health threat globally. In the United States, the prevalence of CVDs in adults was 48.6% in 2020, affecting approximately 127.9 million people [6]. Globally, CVDs caused an estimated 19.05 million deaths in 2020, a 19% increase since 2010, with 607.64 million cases reported worldwide [6]. Heart disease and stroke now claim more lives each year than cancer and chronic lower respiratory disease combined, highlighting the significant burden and severity of CVDs [6]. Given the significant impact of CVDs and the large number of professionals in this field, exploring the applications of ChatGPT is both timely and essential. To do this, we conducted a thorough search of the PubMed and Scopus databases using combinations of the keywords ‘ChatGPT’ or ‘large language model’ with terms such as ‘heart’, ‘cardiovascular’, ‘cardiac’, or ‘cardiology’. As ChatGPT was publicly released on 30 November 2022, our focus was on articles published after this date, excluding non-English articles and those without full-text availability. Based on the analysis of the retrieved literature, this scoping review aims to provide the cardiovascular community with a comprehensive overview of ChatGPT’s innovative applications in cardiovascular disease management. It also addresses current challenges, explores future directions for the development of ChatGPT in cardiovascular medicine, and offers insights into optimizing its use to improve patient outcomes and advance the field (Figure 1). As AI continues to permeate every aspect of healthcare, only by fully understanding both the capabilities and limitations of ChatGPT can the cardiovascular community maximize its benefits, drive innovation, enhance patient care, and advance cardiovascular medicine.

## 2. ChatGPT’s Rapid Rise and Predominance in Public Medical AI Applications

Large language models utilize sophisticated AI algorithms to produce text that mimics human language. Trained on vast datasets sourced from the internet, these models excel in answering questions, summarizing information, translating text, and generating creative content [7]. By inputting specific keywords or queries, users can prompt LLMs to generate text on a wide range of topics in various styles. Notable advancements in LLMs include the development of Google’s Bidirectional Encoder Representations from Transformers and OpenAI’s Generative Pre-trained Transformer (GPT) series. OpenAI released GPT-1 in 2018, followed by GPT-2 and GPT-3 in 2020, with each iteration showcasing enhanced capabilities. In November 2022, OpenAI introduced ChatGPT, an updated LLM that quickly gained significant attention due to its public accessibility, ease of use, and human-like responses achieved through reinforcement learning from human feedback (RLHF) [8]. ChatGPT’s rapid user base growth, exceeding 100 million active users within two months of launch, underscores its widespread adoption. Its popularity as the most widely used LLM today is due to its ability to provide detailed and accurate responses, appealing to students, professionals, and the general public. A study on public perception attributes ChatGPT’s high acceptance rate to its intuitive interface and seamless integration into everyday tasks [9]. Furthermore, the rapid development of ChatGPT, particularly with the advent of GPT-4 and subsequent iterations such as GPT-4o and GPT-4o1-preview, has significantly enhanced its capabilities. These technological advancements have established large language models as versatile and efficient AI tools with the potential to solve complex scientific challenges, including those in the medical field [10].

In the medical field, ChatGPT distinguishes itself for several notable reasons. Its performance on medical examinations, such as the United States Medical Licensing Exam (USMLE), has been rigorously assessed, showing results at or near the passing threshold, even without specialized training. A recent study demonstrates that ChatGPT-4 significantly outperforms its predecessor, ChatGPT-3.5, in answering USMLE Step 2 Clinical Knowledge questions, achieving an accuracy of 87.2% compared to 47.7% [11]. Furthermore, it showcases a 74.6% success rate in generating accurate differential diagnoses from clinical vignettes [11]. The European Exam in Core Cardiology (EECC) is a rigorous postgraduate examination required for the completion of specialty training in cardiology. It consists of 120 multiple-choice questions (MCQs) covering pathophysiology, clinical reasoning, and guideline-recommended medical management, with a typical pass mark of around 60%. In a study, ChatGPT was tested on the EECC using 362 publicly available MCQs. It achieved an overall accuracy of 58.8%, suggesting that it has acquired a broad cardiovascular medicine knowledge base similar to that of cardiologists-in-training [12]. These findings highlight ChatGPT’s potential as both an educational tool and a decision-support system in clinical settings [13]. Comparative analyses with other LLMs, such as Google’s Bard and Meta’s Large Language Model Meta AI (LLaMA), indicate that ChatGPT provides more comprehensive and accurate responses to medical inquiries and tasks [5,14]. Research also shows that ChatGPT provides more accurate, complete, and reproducible answers to home blood pressure monitoring knowledge compared to Bing [15], indicating its value for patients seeking information. This superiority is largely attributed to ChatGPT’s extensive training on diverse datasets and the implementation of RLHF, which enhances the model’s ability to generate credible and contextually appropriate outputs [5]. Furthermore, fine-tuning models, such as ClinicalGPT, with domain-specific data has exhibited markedly enhanced performance in specialized medical tasks, reflecting a higher level of expertise and precision [16]. Additionally, there is a significant increase in the number of medical professionals using ChatGPT, paralleled by a growing trend of patients turning from traditional search engines such as Google to ChatGPT [17]. Compared to traditional search engines, ChatGPT offers a more engaging interactive experience, uses easily understandable language, saves time, and reduces information overload [18].

In conclusion, the evolution of LLMs has greatly advanced multiple fields, with ChatGPT leading due to its exceptional performance, wide applicability, low entry barriers, and high acceptance. Its impact is particularly significant in medicine [19]. As CVDs remain the most widespread and severe health threat globally, ChatGPT’s ability to enhance health education and clinical management in this area is increasingly acknowledged.

## 3. Enhancement of Clinical Decision-Making and Diagnostics in Cardiovascular Medicine with ChatGPT

ChatGPT demonstrates the potential to revolutionize clinical decision-making and diagnostics in cardiovascular medicine by leveraging its ability to analyze patient-reported symptoms, assess risk factors, and support differential diagnoses through extensive medical knowledge databases. Its capabilities in real-time data collection and emergency decision-making can enhance diagnostic accuracy and expedite treatment plans. These attributes underscore its promise in augmenting the diagnostic process and clinical decision-making in cardiovascular diseases [20]. 

A study evaluated ChatGPT’s accuracy in answering cardiovascular trivia questions and providing recommendations for clinical case vignettes [21]. The research involved testing ChatGPT with 50 trivia questions and 20 clinical cases, comparing its responses to expert opinions. ChatGPT correctly answered 92% of trivia questions and matched the actual clinical advice in 85% of case vignettes, with substantial improvement over earlier versions. The findings suggest that ChatGPT has potential as an AI-assisted decision support tool. However, the study’s findings are based on a relatively small sample size and a limited set of clinical cases, which may not fully capture the complexity of real-world cardiovascular scenarios. Therefore, while these results indicate potential, they should be interpreted with caution, as further testing and validation in larger, more diverse clinical settings are necessary to confirm ChatGPT’s effectiveness and reliability as a decision-support tool in cardiovascular care. Adil Salihu et al. evaluated the ability of ChatGPT-4 to improve clinical decision-making and enhance the efficiency of heart teams (HTs) in managing severe aortic stenosis [22]. Data from 150 patients presented at HT meetings were retrospectively analyzed using a standardized multiple-choice questionnaire with 14 key variables. The AI-generated decisions were compared to those made by the HT, with ChatGPT showing a 77% overall agreement. Specifically, agreement rates were 90% for transcatheter valve implantation and 65% for both surgical valve replacement and medical treatment. The study suggested that ChatGPT could potentially support HT decision-making by acting as a second opinion and identifying potential discrepancies. However, human oversight is crucial, and ChatGPT’s effectiveness is highly dependent on the quality of data input [22]. By potentially serving as a second opinion, ChatGPT may improve decision-making efficiency, ensuring comprehensive patient evaluations and enhancing overall care quality in the management of valvular heart diseases. Another study employed a simulated cardiology consultation by asking ChatGPT ten hypothetical questions related to typical clinical scenarios of cardiovascular disorders [23]. The responses were evaluated by medical specialists for accuracy and applicability. Out of the ten scenarios, ChatGPT correctly diagnosed eight and provided appropriate, albeit general, management plans for all. This suggests that ChatGPT can assist in symptom analysis, risk assessment, and preliminary management planning. However, the study also highlighted limitations, such as the need for detailed clinical information and the inability to personalize treatment plans fully [23]. A retrospective study compared the diagnostic accuracy of ChatGPT to that of resident physicians in an emergency department (ED) setting [24]. The study involved 100 adults admitted to the ED with internal medicine issues. Diagnoses made by ED resident physicians and ChatGPT models were compared against final hospital discharge diagnoses using a point system for grading accuracy. Results showed that GPT-4 outperformed both GPT-3.5 (*p* < 0.001) and ED resident physicians (*p* = 0.01) in diagnostic accuracy. GPT-4 demonstrated significant superiority in diagnosing cardiovascular (*p* = 0.03) and endocrine or gastrointestinal diseases (*p* = 0.01) compared to resident physicians and GPT-3.5, respectively. Although the study was retrospective and had a limited sample size, the findings underscore the potential of GPT-4 as a supportive diagnostic tool in ED settings. Moreover, a study investigated the potential of ChatGPT in complementing patient-centered care for heart failure (HF) [25]. Using 30 questions related to HF diagnosis, management, and prognosis derived from online patient forums and physician experience, the responses from ChatGPT were evaluated by two independent HF experts. ChatGPT provided accurate answers in 90% of cases and exhibited a high consistency rate of 93%. Concordance between the two experts’ evaluations was 83%. The study concludes that ChatGPT holds significant promise in enhancing HF diagnosis and patient empowerment, but its occasional omissions of contemporary therapies, such as sodium-glucose cotransporter-2 inhibitors, highlight limitations that may affect its reliability in more nuanced cases. 

ChatGPT’s capabilities have extended to providing support in clinical pharmacy practices, demonstrating its potential to assist clinicians in medication decision-making and management. A study aimed to evaluate ChatGPT’s performance in key domains of clinical pharmacy practice, including prescription review, patient medication education, adverse drug reaction (ADR) recognition, ADR causality assessment, and drug counseling [26]. Real clinical cases and clinical pharmacist competency assessments were used to collect questions and answers, which were then inputted into ChatGPT. Five licensed clinical pharmacists independently rated the responses on a scale from 0 (completely incorrect) to 10 (completely correct). The mean scores of ChatGPT and clinical pharmacists were compared. ChatGPT performed well in drug counseling (8.77 vs. 9.50, *p* = 0.0791) but showed weaknesses in prescription review (5.23 vs. 9.90, *p* = 0.0089), patient medication education (6.20 vs. 9.07, *p* = 0.0032), ADR recognition (5.07 vs. 9.70, *p* = 0.0483), and ADR causality assessment (4.03 vs. 9.73, *p* = 0.023) compared to the pharmacists. Overall, while ChatGPT shows potential as a supplementary tool in drug counseling, it requires substantial improvements to handle complex tasks effectively. Another study further supports ChatGPT’s utility in polypharmacy management for geriatric care. The study evaluated ChatGPT’s performance in deprescribing decisions using standardized clinical vignettes [27]. ChatGPT’s responses demonstrated a consistent internal logic similar to general practitioners, effectively managing polypharmacy by recommending deprescribing based on patients’ activities of daily living status and CVD history. These findings suggest that ChatGPT can provide valuable clinical support in polypharmacy management, highlighting its potential to assist primary care physicians in managing complex medication regimens.

ChatGPT has demonstrated notable potential to enhance diagnostic processes across key cardiovascular diagnostic methods, including echocardiography, electrocardiography (ECG), and cardiovascular magnetic resonance (CMR). ChatGPT has been employed to enhance diagnostic accuracy and interpretation in generating echocardiographic reports [28]. It significantly accelerates the reporting process by producing detailed and concise descriptions of cardiac conditions, summarizing data, and recommending further tests or treatments. By translating complex medical findings into layman’s terms for patients while maintaining technical accuracy for healthcare professionals, ChatGPT improves communication. Furthermore, it ensures consistency and standardization in report generation, reducing variability and subjectivity, which is crucial for high-quality patient care and clinical outcomes [28]. A study evaluated the diagnostic accuracy of ChatGPT-4 in assessing ECG data, comparing its performance to that of emergency medicine specialists and cardiologists [29]. Using 40 ECG cases, ChatGPT-4 demonstrated superior performance in everyday ECG questions compared to both emergency medicine specialists and cardiologists. In more challenging ECG questions, ChatGPT-4 outperformed emergency medicine specialists but performed similarly to cardiologists. Overall, ChatGPT-4 was more successful than both groups in total ECG question accuracy. These findings demonstrate ChatGPT-4’s superior diagnostic accuracy in ECG assessment compared to medical specialists and underscore its potential to enhance clinical decision-making. ChatGPT can also enhance clinical diagnostics by transforming complex CMR reports into more understandable text, thereby improving communication between clinicians and patients. A study evaluated the performance of ChatGPT-4 in simplifying 20 CMR reports, generating three versions of each report for layperson comprehension [30]. Two cardiovascular radiologists assessed the factual correctness, completeness, and lack of potential harm, while 13 laypersons evaluated understandability using a Likert scale. The results showed that ChatGPT-4-generated reports were significantly easier to understand, with a lower Automated Readability Index score compared to original reports. Radiologists rated the ChatGPT-4 reports highly for correctness, completeness, and safety. The study concluded that ChatGPT-4 can reliably simplify CMR reports, ensuring patients receive clear and accurate information. This improved clarity can enhance patient understanding and engagement, leading to more informed clinical decisions and better adherence to treatment plans, ultimately improving cardiovascular care.

While ChatGPT demonstrates considerable potential in clinical decision-making and diagnostics, it also encounters significant challenges, including inaccuracies, hallucinations, and limitations influenced by language and context. A study evaluating ChatGPT’s performance in diagnosing retinal vascular diseases using 1226 fundus fluorescein angiography reports in Chinese highlighted the impact of language on diagnostic effectiveness [31]. Results indicated higher performance with English prompts (F1-score of 80.05%) compared to Chinese prompts (F1-score of 70.47%), though both were lower than ophthalmologists (89.35%) but close to interns (82.69%). English prompts also produced more accurate and complete reasoning steps with fewer errors, underscoring the need for robust models in non-English clinical applications. Moreover, ChatGPT’s application in cardiovascular diagnostics carries significant risks. A case report detailed a delayed diagnosis of a transient ischemic attack due to reliance on ChatGPT’s erroneous evaluation [32]. A 63-year-old man consulted ChatGPT after pulmonary vein isolation, which led to the misclassification of symptoms and a 24-h delay in seeking urgent medical attention. This case emphasizes the critical need for caution and further scrutiny when integrating AI into clinical practice, as errors may result in life-threatening consequences. Additionally, a study on ChatGPT’s performance in complex clinical scenarios involving the American Heart Association’s Advanced Cardiovascular Life Support Guidelines found suboptimal accuracy in following the guidelines for bradycardia and cardiac arrest [33]. Across 20 simulation attempts, ChatGPT’s median accuracy was 69% for cardiac arrest and a notably low 42% for bradycardia. The study identified variability in the outputs of recommended steps, persistent omissions of critical steps, repetitive overemphasis of specific actions such as checking heart rhythm and resuming cardiopulmonary resuscitation (CPR), and erroneous medication information. These findings highlight the need for improvements to ensure consistent and reliable guidance from ChatGPT in clinical settings.

In conclusion, while ChatGPT shows potential to enhance clinical decision-making and diagnostics in cardiovascular medicine, it also faces challenges such as inaccuracies and language-dependent limitations. Addressing these issues is crucial to fully realize its potential as a reliable and effective tool in improving patient outcomes and supporting clinicians in complex medical scenarios.

## 4. Innovative Medical Education for Cardiovascular Professionals and Patients with ChatGPT

The integration of advanced ChatGPT in the CVD field has the potential to revolutionize both professional training and patient education. By providing real-time, accurate, and easily understandable information, ChatGPT bridges the gap between complex medical knowledge and practical application. Its capacity to interpret extensive guidelines, deliver precise clinical answers, and simplify medical jargon ensures that healthcare professionals are well-informed, so patients are better equipped to manage their health.

ChatGPT has already played a significant role in medical professional education and has gained considerable acceptance. An exploratory survey involving 844 participants from various academic and healthcare roles, with a response rate of 49.7%, revealed that 40% of the audience had tried ChatGPT, with more trainees having used it compared to faculty [34]. Those who had used ChatGPT expressed greater interest in utilizing it across a wider range of contexts in the future. The survey highlighted differing perspectives based on roles, with trainees showing more enthusiasm for its use in education, healthcare, and research. Despite some uncertainty, particularly in educational contexts, the broad acceptance and interest indicate that ChatGPT is already making a substantial impact on medical professional education and training. ChatGPT can aid in the education and training of medical professionals by providing clear and concise explanations of complex and extensive guidelines, enhancing their understanding and management. A study evaluated ChatGPT’s ability to answer frequently asked questions (FAQs) and guideline-based questions about acute coronary syndromes (ACS) using the 2023 European Society of Cardiology Guidelines [35]. The accuracy and reproducibility of ChatGPT’s responses were assessed by two cardiologists with ten years of experience. The results showed that 90.3% of ChatGPT’s answers to FAQs scored the highest accuracy and proficiency, with no responses scoring the lowest. For guideline-based questions, 88.3% of responses achieved the highest score. ChatGPT’s answers were also highly reproducible, with 94.4% for FAQs and 90.9% for guideline questions. A cross-sectional study assessed the accuracy and comprehensiveness of chatbot-generated responses to physician-developed medical queries across 17 specialties [36]. Thirty-three physicians generated 284 questions, classified as easy, medium, or hard. ChatGPT responses were scored for accuracy and completeness using Likert scales. The median accuracy score was 5.5 out of 6, and the median completeness score was 3 out of 3. Accuracy scores improved significantly over time and between different versions of the chatbot (GPT-3.5 and GPT-4). This study demonstrated ChatGPT’s high accuracy and capability to provide largely accurate and comprehensive answers to diverse medical queries, underscoring its potential as a valuable educational tool for medical professionals. Given these findings, ChatGPT can be integrated into medical education to provide precise answers to clinical questions, thereby enhancing learning, and facilitating continuous education and professional development. Furthermore, ChatGPT has the potential to provide information on CPR by generating tailored responses based on CPR guidelines [37]. ChatGPT’s responses were compared to traditional methods of obtaining CPR information. The AI demonstrated the ability to quickly provide accurate information, aiding healthcare professionals in emergencies. This study underscores ChatGPT’s potential as an educational tool for enhancing CPR training and emergency response techniques, crucial for preparing healthcare professionals to handle cardiac emergencies effectively.

ChatGPT can enhance patient education by providing understandable, accessible, and empathetic health information. By simplifying medical jargon, it helps patients better understand their conditions and treatment options, leading to improved health literacy, adherence to treatment plans, and informed decision-making. A study evaluated ChatGPT’s ability to provide quality and empathetic responses to patient questions on a public social media forum, comparing its performance to that of verified physicians [38]. Using 195 randomly selected patient-physician exchanges, ChatGPT’s responses were rated higher in both quality and empathy by a team of licensed healthcare professionals. This research highlights ChatGPT’s ability to simplify complex medical information, demonstrating effective patient communication and personalized health education. Building on this, another pilot study investigated whether ChatGPT could improve the readability of online aortic stenosis patient education materials [39]. The study gathered 21 patient education materials from professional cardiothoracic surgical societies and academic institutions in the USA. These materials, originally written at a 10th–12th-grade reading level, were inputted into ChatGPT with the prompt “translate to 5th-grade reading level.” The results showed that ChatGPT-3.5 successfully improved readability to the 6th–7th-grade level across all measures, making complex medical information more accessible and supporting better patient understanding and engagement in managing cardiovascular health. Moreover, ChatGPT has demonstrated its ability to offer comprehensive guidance on hypertension management [40]. Further emphasizing ChatGPT’s potential, a study evaluated ChatGPT’s effectiveness in addressing queries related to lifestyle-related diseases and disorders, enhancing patient engagement and health literacy [41]. Twenty cases, each with four questions, were presented to ChatGPT, and two physicians rated the responses for accuracy and guidance. The study concluded that ChatGPT provides reasonably accurate and useful information, highlighting its potential as a virtual telehealth agent for lifestyle-related diseases. A recent study also confirmed that GPT-4 has shown promising potential in automated medical consultation for cardiovascular diseases, with medical accuracy comparable to human experts [42]. Additionally, ChatGPT’s capability to answer laypeople’s questions about cardiac arrest and CPR was assessed in a comprehensive study [43]. The answers provided by ChatGPT were evaluated by professionals and laypeople for accuracy, relevance, clarity, comprehensibility, and overall value. The study found that ChatGPT’s answers received high ratings for clarity, relevance, accuracy, and comprehensiveness, indicating its potential to enhance public medical education about cardiac arrest.

In summary, ChatGPT shows potential in professional and patient education by providing accessible and comprehensive health information. While it enhances medical training and patient engagement, its effectiveness varies, and challenges with accuracy and reliability persist, requiring further refinement. Nevertheless, it is emerging as a critical instrument in the advancement of medical education.

## 5. Advancements in Cardiovascular Medical Research and Scholarly Communication with ChatGPT

ChatGPT is becoming essential in cardiovascular research and academic writing. This advanced model is transforming how researchers gather, analyze, and communicate data, enhancing predictive modeling, automating literature reviews, and streamlining the writing process. By simplifying complex data analysis and facilitating accurate interpretation, ChatGPT is improving the efficiency and precision of cardiovascular research and scholarly communication.

ChatGPT-4 has the potential to streamline automated machine learning for clinical studies, as its Advanced Data Analysis feature can make machine learning more accessible in medicine by simplifying complex data analyses [44]. Using data from 2142 patients and controls to identify those at risk for cardiac amyloidosis, ChatGPT-4 autonomously selected and optimized machine learning models, employing techniques such as gradient boosting and random forest classifiers. It utilized data preprocessing strategies such as median imputation and standard scaling, followed by 5-fold cross-validation for model validation. The model achieved an area under the receiver operating characteristic curve of 0.954, indicating high diagnostic accuracy [44]. This highlights ChatGPT-4’s significant value in advancing medical research and clinical practice through efficient and precise data analysis. Mohammed Ali et al. conducted a study aimed to explore the influence of the Social Vulnerability Index (SVI) and Digital Literacy (DL) on CVD mortality across US counties using ChatGPT-4 [45]. The researchers integrated data from the Centers for Disease Control and Prevention Wide-ranging Online Data for Epidemiologic Research and the US Census Bureau, identifying significant correlations between the SVI, DL, and CVD mortality through regression modeling. The findings highlighted that incorporating DL and SVI improves the accuracy of mortality predictions, underscoring the importance of these variables in public health research. ChatGPT-4 played a crucial role by identifying relevant variables, generating Python code for data analysis, formulating hypotheses, creating predictive models, and interpreting results, thereby demonstrating its efficiency in handling complex datasets and providing valuable insights. Moreover, ChatGPT has shown potential in cardiovascular imaging research [46]. Utilizing its data analyst functionality, ChatGPT enables researchers to handle descriptive statistics, intergroup analysis, and correlation analysis with high consistency and superior analytical efficiency compared to traditional biostatistical software such as SAS, SPSS, and R. By generating Python code for data integration, hypothesis formulation, and statistical tests, ChatGPT allows researchers with limited programming skills to perform complex data analyses. Its capabilities in automatic data cleaning and visualization streamline the research process, enhancing focus on data interpretation. This functionality demonstrates ChatGPT’s ability to lower operational barriers, making sophisticated data analysis accessible to a broader range of researchers, thereby advancing the field of cardiovascular research. Additionally, ChatGPT can enhance research efficiency by automating data extraction processes. For instance, ChatGPT was utilized to extract procedural details from free-text reports on mechanical thrombectomy in patients with ischemic stroke [47]. The research compared the performance of GPT-4 and GPT-3.5 in accurately extracting data from these reports. ChatGPT-4 demonstrated a higher accuracy rate, successfully extracting 94.0% of data points from internal reports and 90.5% from external reports without the need for further postprocessing. Thus, ChatGPT can significantly reduce manual labor and minimize errors in data collection for prospective studies. Furthermore, ChatGPT has proven effective in automating the screening process for systematic reviews and meta-analyses in medical research. A study evaluated ChatGPT’s efficacy in screening 1198 abstracts from three radiology subfields [48]. Researchers compared ChatGPT’s performance to that of general physicians (GPs) using metrics such as sensitivity, specificity, positive predictive value (PPV), negative predictive value (NPV), and workload savings. ChatGPT completed the screening within an hour, achieving a sensitivity of 95% and an NPV of 99%, slightly outperforming the GPs’ consensus. Additionally, ChatGPT demonstrated low false-negative counts and high workload savings (40–83%). Although ChatGPT had lower specificity and PPV compared to human raters, its high sensitivity and efficiency suggest that it could be a valuable first-line screening tool. Another study confirmed these findings, comparing ChatGPT to human researchers in identifying relevant studies for systematic reviews on mHealth interventions for medication adherence in ischemic stroke patients [49]. This study found that ChatGPT significantly reduced the time required for study identification, demonstrating substantial efficiency. Although human researchers showed higher precision (0.86 vs. 0.77) and relevance percentage (9.8% vs. 3%), ChatGPT identified studies much faster and retrieved a higher number of relevant studies overall.

Generative AI tools, including ChatGPT, have become widely used in the field of academic writing. A recent study reviewed the guidelines of the top 25 cardiology and cardiovascular medicine journals regarding the use of generative AI in scientific writing [50]. The study found that all journals permitted the use of AI tools with specific limitations, such as prohibiting AI from being listed as authors or used for image generation and requiring all authors to take full responsibility for their work. The use of AI in the peer review process is strictly prohibited. Researchers can use ChatGPT for writing as long as they take intellectual ownership and ethical responsibility for its content [51]. AI significantly aids the writing process [52]. For instance, ChatGPT played a pivotal role in a manuscript investigating the long-term administration of proton pump inhibitors (PPIs) and their potential link to adverse cardiovascular outcomes [53]. This research, which reviewed existing systematic reviews and meta-analyses, aimed to determine if prolonged PPI use is associated with major adverse cardiovascular events. ChatGPT was instrumental in generating search strings, screening titles and abstracts, extracting data, summarizing studies, and performing qualitative synthesis and risk of bias assessments. By automating and streamlining these stages, ChatGPT enhanced the efficiency and consistency of the writing process. The study demonstrated that ChatGPT holds significant promise as a tool for evidence synthesis, improving the quality and speed of academic manuscript creation. Furthermore, a case report on the diagnosis of ventricular septal rupture using point-of-care ultrasound in a patient with a history of acute myocardial infarction applied ChatGPT for data collection, literature review, and grammar correction [54]. The authors demonstrated that ChatGPT effectively assisted in gathering precise data, conducting comprehensive literature reviews, and ensuring grammatical accuracy. This highlights ChatGPT’s utility in medical case report writing by enhancing the efficiency and quality of the documentation process.

In summary, ChatGPT has proven to be a transformative tool in cardiovascular research and academic writing. The incorporation of ChatGPT in these areas not only improves efficiency and accuracy but also ensures high-quality and reliable scientific outputs, ultimately contributing to the advancement of cardiovascular medicine.

## 6. Challenges and Future Directions in Integrating ChatGPT into Cardiovascular Medicine

As discussed previously, ChatGPT has shown potential in enhancing cardiovascular medicine but faces significant challenges, including common digital-era issues such as privacy and data security, as well as unique problems specific to its application.

Despite ChatGPT being one of the most advanced large language models in medical applications, a significant concern remains its potential for inaccuracies and hallucinations—instances where the model generates information that appears plausible but is entirely fabricated or incorrect. As highlighted in previous sections and supported by various studies, the error rates in different medical scenarios can range from 5% to 50%, presenting a substantial risk. While human errors are also prevalent, the mistakes made by ChatGPT are often harder to detect and correct, even fooling scientists [55]. ChatGPT’s errors can escalate over time, as its subsequent responses often rely on and amplify earlier inaccuracies, fabricating details or references in the process. This cascading effect not only poses a serious threat to patient safety but also compromises the reliability of scientific research and academic communication [19,56]. A notable example is the use of AI-generated imagery from DALL·E, another OpenAI tool, in CVD education. A study evaluating DALL·E images for congenital heart diseases found significant inaccuracies: 80.8% of images were anatomically incorrect, 85.2% had incorrect text labels, and 78.1% were deemed unusable for educational purposes [57]. This example underscores the broader challenges faced by AI applications in producing accurate and reliable content, further validating concerns about the potential risks associated with AI-generated outputs in medical contexts. Furthermore, compared to the extensive research on ChatGPT’s positive applications, studies focusing on the extent and severity of these risks are limited, making the full scope and seriousness of these issues not yet fully understood.

In addition to accuracy concerns, another significant issue is the fairness and potential biases in AI-generated content. Bias occurs when computer systems systematically and unfairly discriminate against certain groups, particularly underrepresented populations [58]. Among the various types of bias, gender, and racial biases have attracted considerable attention. A study evaluating gender and racial biases in content from seven large language models, including ChatGPT, identified substantial biases [58]. Although ChatGPT exhibited the lowest level of bias and was the only model capable of declining to respond to biased prompts, these findings indicate the ongoing need for improvement in mitigating bias. A non-peer-reviewed study from Stanford University suggested that ChatGPT-3.5 may exhibit gender and racial biases in managing ACS. The study observed that when patients were specified as female, African American, or Hispanic, the frequency of guideline-recommended interventions such as coronary angiography and high-intensity statin prescriptions appeared to decrease. These preliminary findings indicate potential biases within the AI model that could have implications for cardiovascular care outcomes [59].

Furthermore, the application of ChatGPT in the medical field raises significant ethical risks and challenges, which include concrete concerns related to data privacy, intellectual property contamination, and transparency of model decision-making processes [60]. One major issue is the potential violation of data privacy, as ChatGPT and similar models are trained on massive datasets that may inadvertently include identifiable patient information. Without stringent safeguards, there is a risk that sensitive medical data could be exposed or misused. The critical issue is the potential violation of data privacy regulations, especially concerning the protection of Protected Health Information (PHI) under the Health Insurance Portability and Accountability Act (HIPAA) [61]. Generative AI tools such as ChatGPT are not always classified as business associates under HIPAA when processing PHI, which introduces significant legal ambiguities and compliance challenges. For instance, if patients share their health information directly with AI systems, this data may not fall under HIPAA protections, leaving it vulnerable to misuse or re-identification through data triangulation techniques. This situation is further complicated by the lack of explicit guidelines from the U.S. Food and Drug Administration for regulating AI applications such as ChatGPT in healthcare settings [61]. Alongside these privacy concerns, ethical challenges also arise, particularly about ChatGPT’s influence on moral decision-making and academic integrity. A study demonstrated that ChatGPT lacks a consistent moral stance, often providing contradictory advice on the same ethical issue [62]. This inconsistency can influence the moral judgments of users, who may not fully recognize the extent of this impact. Such influence is particularly concerning in medicine, where ethical reasoning is foundational for healthcare professionals and students. Additionally, ChatGPT’s increasing influence in medical research and academic writing has heightened concerns regarding academic integrity and ethics. As reliance on such models for drafting papers grows, issues such as authorship, plagiarism, and the generation of incorrect scientific information come to the forefront. Addressing these ethical challenges is essential to ensure that ChatGPT’s use does not compromise the moral standards critical to both medical practice and academic integrity.

Lastly, the use of ChatGPT in medical education raises concerns about its impact on critical thinking [5]. While LLMs can aid learning, over-reliance on them may hinder students’ or professionals’ ability to evaluate information critically, a skill vital for distinguishing valuable input from irrelevant or incorrect data. This reliance can extend to medical reasoning, risking a decline in analytical and decision-making skills. Transparent regulation of LLM use in educational institutions is essential to preserve and foster critical thinking abilities.

By addressing these challenges, we can better integrate ChatGPT into medicine. Previous discussions have highlighted potential solutions [63,64]. This paper advances these ideas by proposing specific directions for future research and development to enhance the utility, safety, and ethical application of ChatGPT in cardiovascular medicine.

1. Enhancing Training Data Quality and Real-time Knowledge Integration

Improving ChatGPT’s performance in cardiovascular tasks requires incorporating high-quality, domain-specific texts during training, such as clinical notes, patient medical records, surgical reports, diagnostic results, treatment guidelines, and peer-reviewed literature. Expanding the training datasets to include more specialized medical knowledge and ensuring coverage of diverse countries, languages, ethnicities, and religions can reduce biases and enable ChatGPT to provide more accurate and relevant recommendations. Additionally, enabling the model to source real-time information from the internet and continuously update its knowledge base will ensure it remains current with the latest advancements in cardiovascular care.

2. Exploring Custom Models and Optimizing Precision

Developing customized ChatGPT models tailored to specific subfields within cardiology is a promising research direction. This can be achieved by creating disease-specific models for conditions such as atrial fibrillation and heart failure, as well as function-specific models for tasks such as medication guidance and secondary prevention advice. Customization allows the AI to focus on narrower data sets and contexts, reducing complexity and potential errors. An effective approach to enhance accuracy is integrating ChatGPT with information retrieval technologies, which retrieve relevant and authoritative data in real time. This integration not only narrows the focus of the AI model, reducing the likelihood of hallucinations, but also ensures that responses are based on the most current and precise information available [65]. Advanced research into prompt engineering, which involves refining the way questions and requests are posed to the AI, can also significantly improve the model’s accuracy and effectiveness [66]. By tailoring ChatGPT to specific diseases and functions and employing effective prompt engineering strategies, its application can become more targeted, reliable, and efficient in cardiovascular care.

3. Developing Uncertainty Indicators and Enhancing Human Oversight

In cardiovascular care, it is crucial to have tools that indicate the confidence level of AI recommendations, helping users discern when to trust the AI’s advice and when to exercise caution. Clear signals about the reliability of AI-generated content ensure that ChatGPT is used as a helpful assistant rather than the sole decision-maker. To prevent over-reliance on AI and maintain high-quality clinical decisions, rigorous human oversight is essential. Experts can review initial AI recommendations, using these reviews to train the AI and balance efficiency with accuracy. Moreover, future development should establish strict regulations and legal frameworks to ensure human oversight and accountability. Verification by cardiology experts is indispensable in mitigating errors and validating accuracy, with clear accountability for AI-generated outputs resting with human professionals to ensure reliability and safety in clinical settings.

4. Conducting Rigorous and Expert-supervised Clinical Trials

Conducting high-quality, real-world clinical trials to validate ChatGPT’s use in cardiovascular care is essential. These trials should be meticulously designed and conducted under strict human and expert supervision to ensure the reliability and safety of ChatGPT-assisted interventions. Evaluating the effectiveness, acceptance, and practicality of these interventions in clinical practice is crucial. Key endpoints may include reductions in mortality and morbidity, improvements in treatment adherence, and enhanced patient satisfaction. Rigorous, well-supervised clinical research will provide the necessary evidence to assess ChatGPT’s true impact on cardiovascular disease management. To support these efforts, structured validation tools such as the Artificial Intelligence Performance Instrument (AIPI) offer an effective method for assessing ChatGPT in clinical applications [67]. AIPI exemplifies a consistent framework for evaluating the reliability and validity of ChatGPT’s performance. By utilizing such standardized tools, alongside expert oversight, healthcare settings can ensure AI models maintain accuracy and safety, facilitating their effective and trustworthy integration into clinical practice.

5. Establishing Ethical and Regulatory Compliance Guidelines

Clear ethical guidelines for the use of ChatGPT are necessary, addressing issues of authorship, plagiarism, and accountability for AI-generated content. Transparency in the use of AI in research, clinical practice, and publications is crucial to prevent misuse and ensure ethical compliance. Scholars have suggested key recommendations, including prohibiting ChatGPT from being cited as an author, ensuring that users possess a basic understanding of the models, and restricting these models from generating the entirety of the manuscript text [68]. Proper acknowledgment of AI use is essential to maintain ethical standards and integrity in academic writing. These guidelines should be discussed and unified to form a broad consensus for effective management, thereby upholding the credibility and ethical standards of work involving ChatGPT. To further integrate ChatGPT into medical applications while ensuring compliance with legal and privacy regulations, several strategies must be adopted. Developers should implement HIPAA-compliant frameworks and collaborate with healthcare entities to establish business associate agreements, ensuring the proper regulation of PHI use and processing. De-identification of health data must also be prioritized, using established methods such as the Safe Harbor method or expert determination to minimize the risk of unauthorized access or re-identification [61]. Additionally, proactive adherence to federal guidelines, such as those from the National Institute of Standards and Technology AI Risk Management Framework and Federal Trade Commission regulations, will help maintain legal compliance and protect patient data integrity [61].

By following these five key strategies, ChatGPT’s development can be precisely aligned with the needs of the cardiovascular field, ensuring safe, ethical, and effective applications in patient care, research, and education. Strengthening its core capabilities and integrating it with complementary AI technologies, such as advanced machine learning and deep learning, can further enhance its impact [69], driving innovation and expanding its effectiveness across the medical domain.

## 7. Conclusions

ChatGPT has become a transformative tool in cardiovascular medicine, significantly enhancing clinical decision-making, education, and research. Despite facing challenges such as accuracy and ethical considerations, there are clear pathways to address these issues. With ongoing advancements and targeted solutions, the future of ChatGPT in cardiovascular medicine is promising. This technology has already benefited many patients, healthcare professionals, and medical students, and as it continues to evolve, it will further enhance their capabilities and drive greater advancements in the field.

## Data Availability

Not applicable.
